# Allelic Imbalance of mRNA Associated with *α*2-HS Glycoprotein (Fetuin-A) Polymorphism

**DOI:** 10.1155/2015/865053

**Published:** 2015-10-15

**Authors:** Yoshihiko Inaoka, Motoki Osawa, Nahoko Mukasa, Keiko Miyashita, Fumiko Satoh, Yu Kakimoto

**Affiliations:** Department of Forensic Medicine, Tokai University School of Medicine, Isehara, Kanagawa 259-1193, Japan

## Abstract

Alpha 2-HS glycoprotein (AHSG), also designated as fetuin-A, exhibits polymorphism in population genetics consisting of two major alleles of *AHSG* ^*∗*^ *1* and *AHSG* ^*∗*^ *2*. The serum level in the *AHSG* ^*∗*^ *1* homozygote is significantly higher than that of the *AHSG* ^*∗*^ *2* homozygote. This study examined the molecular mechanism for the *cis*-regulatory expression. To quantitate allele-specific mRNA in intra-assays of the heterozygote, RT-PCR method employing primers that were incorporated to the two closely located SNPs was developed. The respective magnitudes of *AHSG* ^*∗*^ *1* to *AHSG* ^*∗*^ *2* in the liver tissues and hepatic culture cells of PLC/PRF/5 were determined quantitatively as 2.5-fold and 6.2-fold. The mRNA expressional difference of two major alleles was observed, which is consistent with that in the serum level. The culture cells carried heterozygous genotypes in rs4917 and rs4918, but homozygous one in rs2248690. It was unlikely that the imbalance was derived from the SNP located in the promotor site. Furthermore, to investigate the effect of mRNA degradation, RNA synthesis in the cell culture was inhibited potently by the addition of actinomycin-D. No marked change was apparent between the two alleles. The results indicated that the *cis*-regulatory expressional difference is expected to occur at the level of transcription or splicing of mRNA.

## 1. Introduction

Alpha 2-HS glycoprotein (AHSG), also designated as fetuin-A, is a 46 kDa serum protein that is synthesized mainly in hepatic cells [1]. The protein is known to exhibit multiple functions [2]. AHSG acts as a natural inhibitor of insulin receptor tyrosine kinase [3]. Goustin and Abou-Samra [4] summarized its molecular mechanism and pathogenesis. AHSG was recently revealed to be an endogenous ligand for Toll-like receptor 4 playing a crucially important role in stimulating adipose tissue inflammation, resulting in insulin resistance [5]. These evidences show the close relation of AHSG to occurrence of type 2 diabetes [6]. Moreover, AHSG is bound to calcium phosphate with high affinity. It is a major component of the extracellular matrix of the bone [7]. The major physiological function is to prevent extracellular calcification [8]. The low plasma level of AHSG from hemodialysis accelerates the stiffening and calcification of arteries [9].

AHSG used to be a conventional genetic marker for use in isoelectric focusing in anthropology [10]. Two major alleles of *AHSG* ^*∗*^ *1* and *AHSG* ^*∗*^ *2* comprise double nonsynonymous substitutions of T230M (rs4917) in exon 6 and T238S (rs4918) in exon 7 [11]. In addition to the amino acid replacement, the plasma AHSG level in the *AHSG* ^*∗*^ *2* homozygote is approximately 20% lower than that of the *AHSG* ^*∗*^ *1* homozygote [12–14]. This phenomenon means that the linked nucleotide substitutions are directly attributable to biosynthesis or degradation of mRNA. In particular, SNP (rs2248690) of AP-1 binding site in the promotor region affects the transcriptional activity [15]. This SNP has been nominated as the most potent association with type 2 diabetes [16]. However, the mechanism of the* cis*-acting expressional differences has not been elucidated.

To assess the abundance of allele-specific mRNA, intraindividual assay of the heterozygote is preferred over interindividual assays, which are subject to several potential environmental errors such as variation in mRNA quality as a result from specimen degeneration [17]. For the intracellular assay, human hepatoma cell lines that have heterozygous AHSG genotype were searched [18]. In order to develop such intra-assays, the specific detection of coexisting alleles in the heterozygote is crucially important, relying on the ability to distinguish one allele from the other with no cross-reaction. Buckland [19] described several molecular procedures to assess allele-specific expressional differences.

In the present study, we developed a novel RT-PCT procedure to quantitate the amounts of two allelic transcripts of* AHSG* separately, in which primers were designed with incorporation to the two closely located SNPs of rs4917 and rs4918. The allelic expression differences in the heterozygote were examined in the liver tissue and the culture cells of hepatic carcinoma cell lines. Finally, to elucidate the effects of degradation, mRNA levels were examined after the addition of actinomycin-D synthesis as a potent inhibitor of RNA synthesis.

## 2. Materials and Methods

### 2.1. Subjects

To examine amounts of AHSG mRNA, four specimens of postmortem liver tissues obtained during autopsy at the Department of Forensic Medicine, Tokai University School of Medicine, were available for mRNA extraction. Then they were stored at −80°C until use [11]. All specimens were heterozygous in the* AHSG* genotype. After being approved by our institutional ethics committee, informed consent was obtained from the family members of deceased subjects. DNA from unrelated individuals was used to ensure the linkage of SNPs. Buccal swabs were obtained from 52 Japanese individuals for DNA extraction, as described previously [20].

### 2.2. Cell Culture

Cells of HepG2, PLC/PRF/5, and HeLa cell lines were cultured in DMEM medium supplemented with penicillin at 100 mg/L, streptomycin at 100,000 U/L and 10% fetal bovine serum (Biological Industries Ltd., Israel) in a humidified atmosphere containing 5% CO_2_ at 37°C. The cells for culture were dispersed in 60 mm dishes with 7.5 × 10^6^ cells in 5 mL of the medium at overnight and were collected for DNA and total RNA extraction. After washing with phosphate buffered saline, the confluent cells were treated in 0.25% w/v trypsin and 1 mM EDTA at 37°C for 5 min, followed by adjustment to 1 × 10^5^ cells in 1 mL of DMEM medium.

To arrest RNA synthesis, the medium was replaced with fresh medium containing 5 *μ*M actinomycin-D (Wako Pure Chemical Industries Ltd., Osaka, Japan). Cells were harvested at 0, 2.5, 5, 7.5, and 10 h after addition of the transcriptional inhibitor. Then they were examined using real-time PCR.

### 2.3. Extraction of DNA and RNA

Homogenized liver tissue of 1 g and the suspended culture cells were dissolved in Trizol solution (Invitrogen Life Technologies, Carlsbad, CA, USA). From 500 *μ*L of the cell lysates, RNA was extracted using as Rneasy Lipid Tissue Mini Kit (Qiagen Inc., Hilden, Germany) according to instructions provided by the manufacturer. Total RNA was reverse-transcribed to cDNA using Superscript III (Invitrogen) priming with oligo-dT combined with T7 RNA polymerase promoter.

DNA was extracted using spin columns of the QIAamp mini kit (Qiagen). Genotyping was performed using PCR-restriction fragment length polymorphism (RFLP), as described previously [11].

### 2.4. Allele-Specific PCR

To detect mRNA expression levels of *AHSG* ^*∗*^ *1* and *AHSG* ^*∗*^ *2* separately in the intra-assays, allele-specific amplification was performed in the real-time PCR procedure. We designed specific oligonucleotide primer sets of 5′-AGGTTGCAGTGACCTGCAC-3′ and 5′-TCTGGTTGGGGCTGTGAGG-3′ for *AHSG* ^*∗*^ *1*, and 5′-AGGTTGCAGTGACCTGCAT-3′ and 5′-TCTGGTTGGGGCTGTGAGC-3′ for *AHSG* ^*∗*^ *2*, in which substitutions of rs4917 and rs4918 are incorporated into the 3′-terminal of both forward and reverse oligonucleotide primers. The amplified fragments were detected using Power SYBER Green PCR Master Mix (Applied Biosystems, Life Technologies) in optical 96-well MicroAmp plates using 7300 real-time PCR system (Applied Biosystems). To generate standard curves, the plasmid vectors recombined by the *AHSG* ^*∗*^ *1* and *AHSG* ^*∗*^ *2* cDNA fragments were used as the template [11]. Fivefold serial dilutions at 0.4, 0.08, 0.016, 0.0032, and 0.00064 ng were added to the reaction mixture of 25 *μ*L, in which 1 ng of the template corresponded to 4.18 × 10^−16^ mol and 2.52 × 10^8^ copies. To confirm the cross-reactivity to the other allele, the allele-specific primer pairs operated against the other allelic vector as the template. For the control, *β*-actin amplification using a primer set of 5′-GGACTTCGAGCAAGAGATGG-3′ and 5′-AAGGAAGGCTGGAAGAGTGC-3′ was employed using pGEMT-Easy plasmid recombined by the PCR fragment.

The critical threshold (Ct) values for each PCR reaction were calculated automatically using the ABI prism sequence detection system (ver. 1.6). The starting concentration of DNA fragment present in each reaction was calculated by comparing Ct values of unknown samples to those of standards with known amounts of target DNA fragment; Ct values were plotted against the log of the initial concentration of DNA to produce a standard curve. The Ct value that is dependent upon the starting copy number of the target is defined as the cycle number at which a statistically significant increase of the reporter fluorescence can first be detected.

The mean of triplicates was selected for each measurement. To compare two groups statistically, data from the experiments were analyzed using Student's *t*-test. Results for which *P* < 0.01 were inferred as significant.

### 2.5. Linkage Analysis

**Figure 1:**
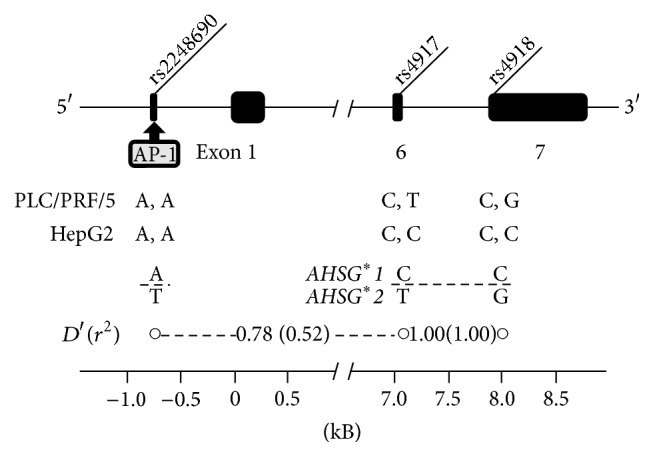
Gene structure of* AHSG* indicating three major SNPs introduced into this study. Genotypes in the culture cell lines of HepG2 and PLC/PRF/5 and their linkage disequilibrium of *D*′ and *r* ^2^ are indicated.

PCR amplification was performed with extracted DNA from each of 52 individuals. The three SNPs examined here were presented in Figure 1. Linkage analysis was conducted using software (SNPAlyze ver. 7 standard; Dynacom Co. Ltd., Yokohama, Japan); *r* square (*r* ^2^) and the linkage disequilibrium (*D*′) were calculated.

## 3. Results

### 3.1. Allele-Specific Detection of mRNA

**Figure 2:**
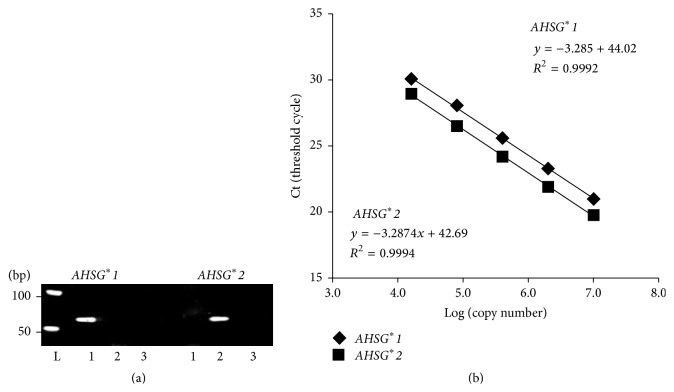
Allele-specific PCR amplification. (a) Nondenaturing polyacrylamide gel electrophoresis of allele-specific RT-PCR products; left: primer set for *AHSG* ^*∗*^ *1* and right: that for *AHSG* ^*∗*^ *2*. Lane 1: recombinant *AHSG* ^*∗*^ *1* fragment for template; lane 2: recombinant *AHSG* ^*∗*^ *2* one for template; lane 3: no template for the negative control. (b) Standard plot for allele-specific real-time PCR amplification. The least-squares regression model was calculated from values of cycle threshold (Ct) versus addition of the recombinant DNA fragment in triplicate.

The first objective was comparison of the relative expression levels of two* AHSG* alleles within the same individual and the single culture cell line. The comparison in these intra-assays of the heterozygote depends on specific detection of the alleles. As Figure 1 shows, the highly linked nucleotide substitutions of rs4917 and rs4918 are closely located in adjacent exons 6 and 7. In the present attempt, these two substitutions are incorporated into the 3′ end of both forward and reverse PCR primers, which yields 61 bp DNA products in length from cDNA. As presented in Figure 2(a), no cross-reactivity was observed using the other template like the *AHSG* ^*∗*^ *1* amplification against *AHSG* ^*∗*^ *2* allelic fragment as the template.

The efficiencies of amplification might be mutually distinct. Therefore, the standard curve was constructed separately to obtain the absolute copy number. However, no apparent difference of fluorescence intensity in the amplification curves was observed in the subcloned DNA fragments of *AHSG* ^*∗*^ *1* and *AHSG* ^*∗*^ *2* as the template. The standard curve was generated by regression in which amplification of the template of recombinant plasmid at various concentrations showed linearity over a range of four orders of magnitude (Figure 2(b)).

### 3.2. Intraindividual Assay

**Figure 3:**
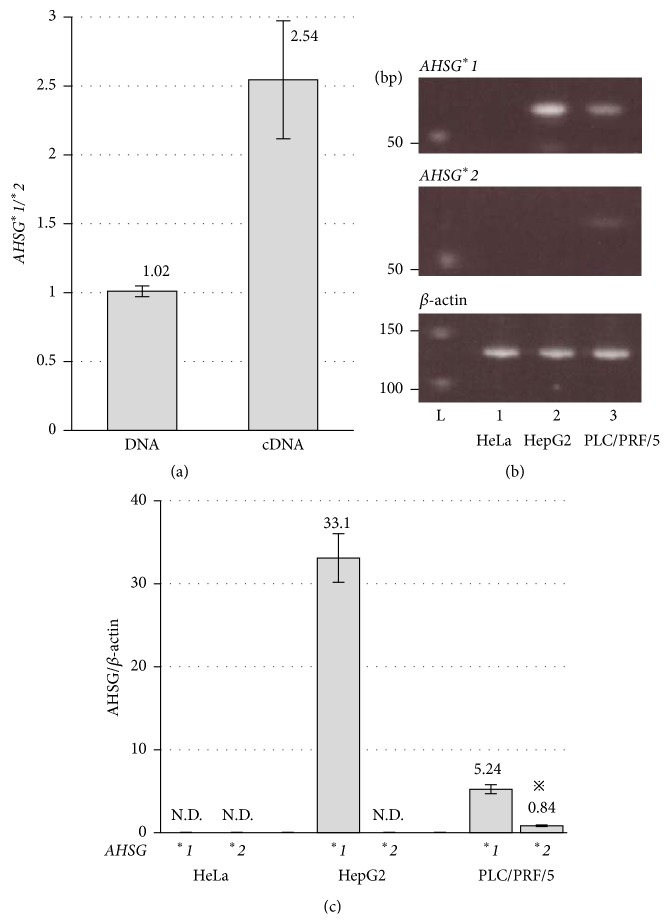
Two intra-assays of intraindividual assay in the human liver tissues (a) and intracellular assay in three culture cell lines (b and c). (a) Relative amount of *AHSG* ^*∗*^ *1* mRNA to that of *AHSG* ^*∗*^ *2* was obtained in genomic DNA (left) and mRNA from the liver tissue (right). Bars represent the mean ± SD of four specimens. (b) Nondenaturing polyacrylamide gel electrophoresis of allele-specific RT-PCR for ^*∗*^ *1*, ^*∗*^ *2*, and *β-actin* products. Lane 1: HeLa; lane 2: HepG2; and lane 3: PLC/PRF/5. (c) Comparison of allelic mRNA amount of *AHSG* ^*∗*^ *1* and *AHSG* ^*∗*^ *2* to *β-actin*. N.D. denotes not detected. Bars represent the mean ± SD in triplicate; ^*※*^ *P* < 0.01 significant versus the amount of *AHSG* ^*∗*^ *1*.

The real-time PCR procedure quantitated the abundance of transcript amounts of *AHSG* ^*∗*^ *1* and *AHSG* ^*∗*^ *2* mRNA in the hepatic tissues from four heterozygous individuals. As depicted in Figure 3(a), a relative amount of ^*∗*^ *1* mRNA was significantly greater from 2.2-fold to 3.2-fold than that of ^*∗*^ *2* with a mean and SD of 2.5 ± 0.4. As the control, the allele-specific PCR amplification was applied to genomic DNA, which caused equal amplification of ^*∗*^ *1* and ^*∗*^ *2*.

### 3.3. Intracellular Assay

The allelic imbalance was evaluated in cells of culture cell lines, as well. Cells of the HepG2 cell line, of which genotyping from the genome sequence revealed *AHSG* ^*∗*^ *1* homozygote, expressed only *AHSG* ^*∗*^ *1* mRNA abundantly but not *AHSG* ^*∗*^ *2* (Figure 3(b)). The HeLa cells did not express* AHSG* mRNA, which served as the negative control. Another hepatic PLC/PRF/5 cell line was genotyped to the heterozygote. Allele-specific amplification revealed that the *AHSG* ^*∗*^ *1* mRNA count was approximately 6.2-fold greater than that of *AHSG* ^*∗*^ *2* (*P* < 0.01) in the PLC/PRF/5 cells (Figure 3(c)).

### 3.4. Effect of Actinomycin-D

**Figure 4:**
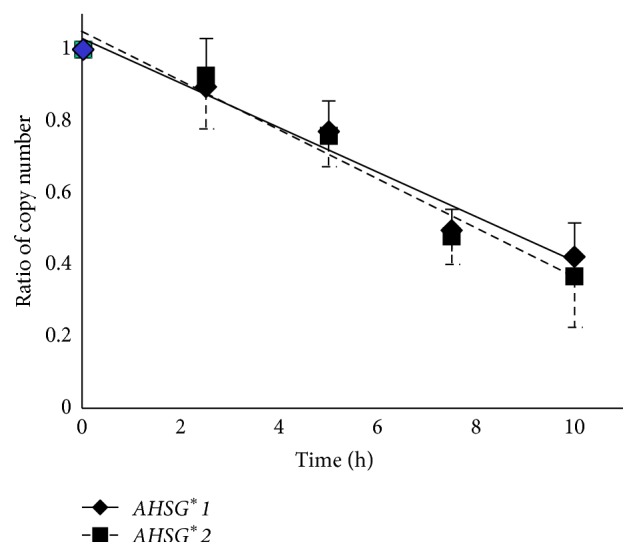
Inhibition of mRNA synthesis by the addition of actinomycin-D. After addition of actinomycin-D, the cells of PLC/PRF/5 were harvested to find mRNA numbers using the allele-specific RT-PCR. Solid and broken lines indicate *AHSG* ^*∗*^ *1* and *AHSG* ^*∗*^ *2*, respectively.

To assess the cause of allelic imbalance of* AHSG* transcripts further, actinomycin-D, which arrests RNA synthesis, was added to the culture medium for the PLC/PRF/5 cells. Cells were harvested at 2.5, 5, 7.5, and 10 h after addition of the transcription inhibitor. Then the mRNA level was quantitated using real-time PCR analysis.  Figure 4 demonstrates the degradation of mRNA as the ratio to the amount at time zero. No significant change was evident in the degradation curves between *AHSG* ^*∗*^ *1* and *AHSG* ^*∗*^ *2* mRNA transcripts, indicating that a major factor that affects the allelic imbalance may be attributable to transcription, but not to degradation.

### 3.5. Linkage Analysis from Genotyping

The A/T SNP at rs2248690 in the promoter regulatory region potentially affects transcriptional activity of* AHSG* [15]. The genotype of rs2248690 in cells of the PLC/PRF/5 cell line was a homozygote of (A, A), although the PLC/PRF/5 cells exhibited the* AHSG* heterozygote. This result indicated no apparent interaction of the allelic imbalance of* AHSG* with the variety of AP-1 binding in the promoter region. Therefore, the linkage between the SNP and the phenotypic determinants of two SNPs (rs4917 and rs4918) was confirmed in the unrelated Japanese individuals. Incomplete linkage was found: the linkage disequilibrium values were 0.78 in *D*′ and 0.52 in *r* ^2^ (Figure 1). Moreover, in the four heterozygous specimens using the intraindividual assay included three homozygotes of (A, A) at rs2248690.

## 4. Discussion

Our previous work demonstrated that the AHSG serum level in *AHSG* ^*∗*^ *1* homozygote is approximately 20% higher than that of the *AHSG* ^*∗*^ *2* homozygote [12]. For many years, association studies in a population group showed that this polymorphism is related to various physical statures such as bone mineral density [21, 22] and leanness [13]. Moreover, a number of association studies with major diseases such as type 2 diabetes [6, 16, 23], lipid levels [24, 25], and ischemic heart disease [26–28] have been reported extensively.

In the present study, to examine the origin of the major phenotypic differences, we developed a quantitative real-time PCR assay for the sensitive and specific detection of the two* AHSG* alleles, where two separated titrations for absolute quantification were necessary to analyze the respective alleles. This series of experiments confirmed a significant difference in the mRNA transcription level of two major alleles.

In the intraindividual assay using liver tissues of the heterozygote, the *AHSG* ^*∗*^ *1* mRNA level was significantly, over two times, higher than that of *AHSG* ^*∗*^ *2* mRNA in the real-time PCR detection. In the intracellular assay using PLC/PRF/5 cells of the heterozygote, ^*∗*^ *1* mRNA level was approximately six times higher than that of ^*∗*^ *2* mRNA as well. It was readily apparent that the AHSG protein level in serum was correlated with mRNA level in the hepatocytes. This result means that some* cis*-acting mechanism operates in the process of protein biosynthesis. A number of other functional* cis*-regulatory variations are known. Among them, allelic differences of twofold or greater in the rate of transcription are common, and even more than 20-fold differences are not uncommon [29, 30].

In the experiment using actinomycin-D that inhibits mRNA synthesis, no apparent difference was observed in the ratio of ^*∗*^ *1*/^*∗*^ *2* allele numbers, suggesting that the allelic imbalance appeared not to be derived from the difference in the degeneration process of mRNA.

In terms of the allelic imbalance of mRNA level, the molecular cause was unknown. However, the possibility exists of linkage of the ^*∗*^ *1* and ^*∗*^ *2* alleles with SNP in the promoter region, in which transcriptional factor AP-1 binding site is present. Inoue et al. [15] demonstrated that SNP at −768 nucleotide position in the AP-1 binding site affects the transcription of AHSG in the* cis*-acting manner. The AP-1 binding type (TGTGTCA) is bound to AP-1 more tightly than the other AP-1 nonbinding type (AGTGTCA). Their luciferase assay showed that tight binding of T-type reduces the transcriptional activity. This SNP (rs2248690) in the promoter region potentially links to *AHSG* ^*∗*^ *1* and *AHSG* ^*∗*^ *2* polymorphism and affects the allelic imbalance of mRNA by changing the transcription. The homozygote of (A, A) at −768 n.p. was observed in the heterozygous cells of PLC/PRF/5, indicating that the promoter SNP associated with AP-1 binding is unlikely to be the main cause for allele-specific imbalance of transcription. We deduce that the allelic imbalance originated from the two SNPs themselves of exons 6 and 7 or other closely linked SNPs within the haploblock.

Because the two critical SNPs of rs4917 and rs4918 are present in the vicinities of exon-intron junctions, the linked substitutions potentially affect splicing of transcripts [31]. To examine another mRNA species caused by alternative splicing, RT-PCR using several primer pairs was performed extensively, but no evidence was obtained (data not shown). Moreover, another extensive search of the Expression Sequence Tag (EST) database at the NCBI site (http://www.ncbi.nlm.nih.gov/nucest/) failed to reveal any potential cDNA fragment to infer the occurrence of alternative splicing. These preliminary results suggest that the allelic imbalance was not caused by alternative splicing.

We have speculated that the imbalance originated from biosynthesis of mRNA or inhibited splicing activity in *AHSG* ^*∗*^ *2*, which reduces the amount of mature mRNA, but the exact mechanism has not been elucidated through this series of experiments. It seems certain that the imbalance derived from the relation with the two major SNPs and other highly linked SNPs, at least.

Although *AHSG* ^*∗*^ *2* has been derived from the older ^*∗*^ *1* allele lately, this ^*∗*^ *2* allele distributes rapidly to all examined population group, as shown previously [32]. This polymorphism is potentially related to natural selection in the human evolution, but its true physiological significance remains unclear.

## Supplementary Material

Genotypes of three SNPs in 52 unrelated individuals.
