# Supplementary material for: Allelic Imbalance of mRNA Associated with α2-HS Glycoprotein (Fetuin-A) Polymorphism
Source: Dis Markers. 2015 Oct 15;2015:865053. doi: 10.1155/2015/865053 (PMC4624919; doi:10.1155/2015/865053)
Supplement: Supplementary file 1 — Genotypes of three SNPs in 52 unrelated individuals. [file 865053.f1.docx]

Supplementary Table. Genotyping of the 52 unrelated individuals

| Number | rs4917(ex6) | rs45918(ex7) | rs2248690 |
| --- | --- | --- | --- |
| 802 | c/c | c/c | a/a |
| 803 | c/c | c/c | a/a |
| 805 | c/c | c/c | a/a |
| 807 | t/c | g/c | a/t |
| 808 | c/c | c/c | a/a |
| 809 | c/c | c/c | a/a |
| 812 | t/c | g/c | a/t |
| 814 | c/c | c/c | a/a |
| 815 | c/c | c/c | a/a |
| 819 | c/c | c/c | a/a |
| 821 | t/c | g/c | a/t |
| 827 | c/c | c/c | a/a |
| 831 | t/c | g/c | a/a |
| 836 | t/c | g/c | a/t |
| 837 | t/c | g/c | a/t |
| 842 | t/c | g/c | a/t |
| 702 | t/c | g/c | a/t |
| 705 | c/c | c/c | a/a |
| 706 | c/c | c/c | a/a |
| 708 | c/c | c/c | a/a |
| 710 | t/c | g/c | a/t |
| 712 | t/c | g/c | a/a |
| 713 | c/c | c/c | a/a |
| 719 | c/c | c/c | a/a |
| 721 | t/c | g/c | a/t |
| 723 | c/c | c/c | a/a |
| 727 | t/c | g/c | a/t |
| 735 | t/c | g/c | a/t |
| 736 | c/c | c/c | a/a |
| 737 | t/c | g/c | a/t |
| 740 | t/c | g/c | a/t |
| 743 | t/c | g/c | a/t |
| 744 | c/c | c/c | a/a |
| 745 | c/c | c/c | a/t |
| 746 | c/c | c/c | a/a |
| 747 | c/c | c/c | a/t |
| 751 | t/c | g/c | a/a |
| 752 | t/c | g/c | t/t |
| 755 | c/c | c/c | a/a |
| 757 | c/c | c/c | a/a |
| 758 | t/c | g/c | a/t |
| 759 | t/c | g/c | a/a |
| 760 | t/c | g/c | a/a |
| 761 | c/c | c/c | a/a |
| 120 | c/c | c/c | a/a |
| 118 | c/c | c/c | a/a |
| 134 | t/t | g/g | a/t |
| 176 | t/t | g/g | a/t |
| 391 | t/c | g/g | a/a |
| 392 | c/c | c/c | a/t |
| 393 | t/c | g/c | a/t |
| 394 | t/c | g/c | a/t |
